# Association between lactate/albumin ratio and 28-day mortality in ICU critical patients with coronary heart disease: a retrospective analysis of the MIMIC-IV database

**DOI:** 10.3389/fcvm.2024.1486697

**Published:** 2024-11-18

**Authors:** Ying Liu

**Affiliations:** ^1^ Department of Radiology, The First Affiliated Hospital, Jiangxi Medical College, Nanchang University, Nanchang, Jiangxi, China; ^2^ Jiangxi Province Medical Imaging Research Institute, Nanchang, Jiangxi, China; ^3^ Clinical Research Center for Medical Imaging, Nanchang, Jiangxi, China

**Keywords:** lactate/albumin ratio, biomarker, coronary heart disease, 28-day mortality, MIMIC-IV database

## Abstract

**Background:**

The serum lactate/albumin ratio (LAR) is commonly employed for monitoring and evaluating the prognosis of critically ill patients. Both elevated lactate levels and decreased albumin levels may reflect the body's stress response and inflammatory reaction. Coronary heart disease (CHD), with common complications including myocardial infarction, arrhythmia, heart failure, is one of the leading causes of global death. Therefore, it is crucial to explore biomarkers that can predict the prognosis and mortality of CHD patients.

**Methods:**

This is a retrospective study in which the data is from the MIMIC-IV database. Our study assessed the association between LAR value and mortality within 28 days of admission in a total of 1,902 CHD patients from the Beth Israel Deaconess Medical Center.

**Results:**

The results demonstrated a significant increase in 28-day mortality among individuals with higher LAR values. Multivariate analysis by Cox proportional hazard model revealed an incremental rise in mortality across each quartile with the increase of LAR value. Furthermore, restricted cubic spline (RCS) Cox regression analysis further revealed that higher LAR values were associated with increased 28-day mortality in the CHD patients. And subgroup analysis confirmed that the LAR level could serve as an independent predictor of 28-day mortality with CHD patients.

**Conclusions:**

Our study demonstrated that the LAR value can be an important risk predictor of 28-day mortality in patients with CHD, and a higher LAR associate with increased mortality rate.

## 1 Introduction

Coronary heart disease (CHD) is one of the most common cardiovascular diseases worldwide, particularly in developed countries and some developing countries where its incidence is relatively high. Additionally, CHD is one of the leading causes of global death (1, 2). According to the World Health Organization, over 17 million people die each year from cardiovascular diseases (including CHD) (3). Acute myocardial infarction, arrhythmias, heart failure, and thrombosis resulting from CHD are major cause of death (4). Therefore, given the high incidence and mortality rates associated with CHD, it holds immense significance to explore biomarkers capable of predicting prognosis or mortality among hospitalized CHD patients.

Lactate is a product of cellular metabolism, particularly in hypoxic conditions. Elevated lactate levels typically indicate tissue hypoxia or hemodynamic disturbances, such as those seen in sepsis, shock, or heart failure (5). Serum albumin is a protein synthesized by the liver and is abundant in blood. It has antioxidant and anti-inflammatory properties, and primarily functions to maintain plasma colloid osmotic pressure, transport various substances such as hormones, fatty acids, and drugs. Low serum albumin levels (<3.5 g/dl) are often associated with poor prognosis, which may suggest malnutrition, chronic disease, acute inflammation or impaired liver function. Studies have revealed that low albumin levels are correlated with higher mortality rates, longer hospital stays, and increased complication rates (6–8). Serum albumin level is considered an important prognostic biomarker for ICU patients (9).

The serum lactate/albumin ratio (LAR) is a clinical indicator used to evaluate status of the patients, particularly those of critically ill patients. A higher ratio typically indicates a poorer prognosis. During acute illness or post-trauma, elevated lactate levels and decreased albumin levels may reflect the body's stress and inflammatory responses. The ratio combines information of both lactate and albumin levels, providing a more comprehensive evaluation of the patient's status (10).

Our study evaluated the relationship between the LAR level and 28-day mortality in CHD patients, and revealed that higher LAR levels associate with higher mortality rates. Therefore, LAR can serve as an independent predictor of higher 28-day mortality in CHD patients.

## 2 Methods

### 2.1 Data source

This is a retrospective study, data from the MIMIC-IV database (version 2.2). This database covers information on all patients admitted to the Beth Israel Deaconess Medical Center (BIDMC) during the years from 2008 to 2019, which includes comprehensive information on patients’ hospital stay, laboratory tests, medication treatments, vital signs, and so no. To protect patient privacy, all personal information has been de-identified and replaced with random codes. Therefore, obtaining patients’ informed consent or ethical approval was not necessary for this study. The MIMIC-IV (v.2.2) database can be downloaded from the online platform PhysioNet (https://physionet.org/content/mimiciv/2.2/). In order to gain access to this database and extract data, Ying Liu, the first author of this study, successfully completed the Collaborative Institutional Training Initiative (CITI) courses “Conflicts of Interest” and “Data or Specimens Only Research” (ID: 42343630). Consequently, we obtained the necessary qualifications for using the database and extract data.

### 2.2 Data selection criteria and exclusion criteria

**Figure 1:**
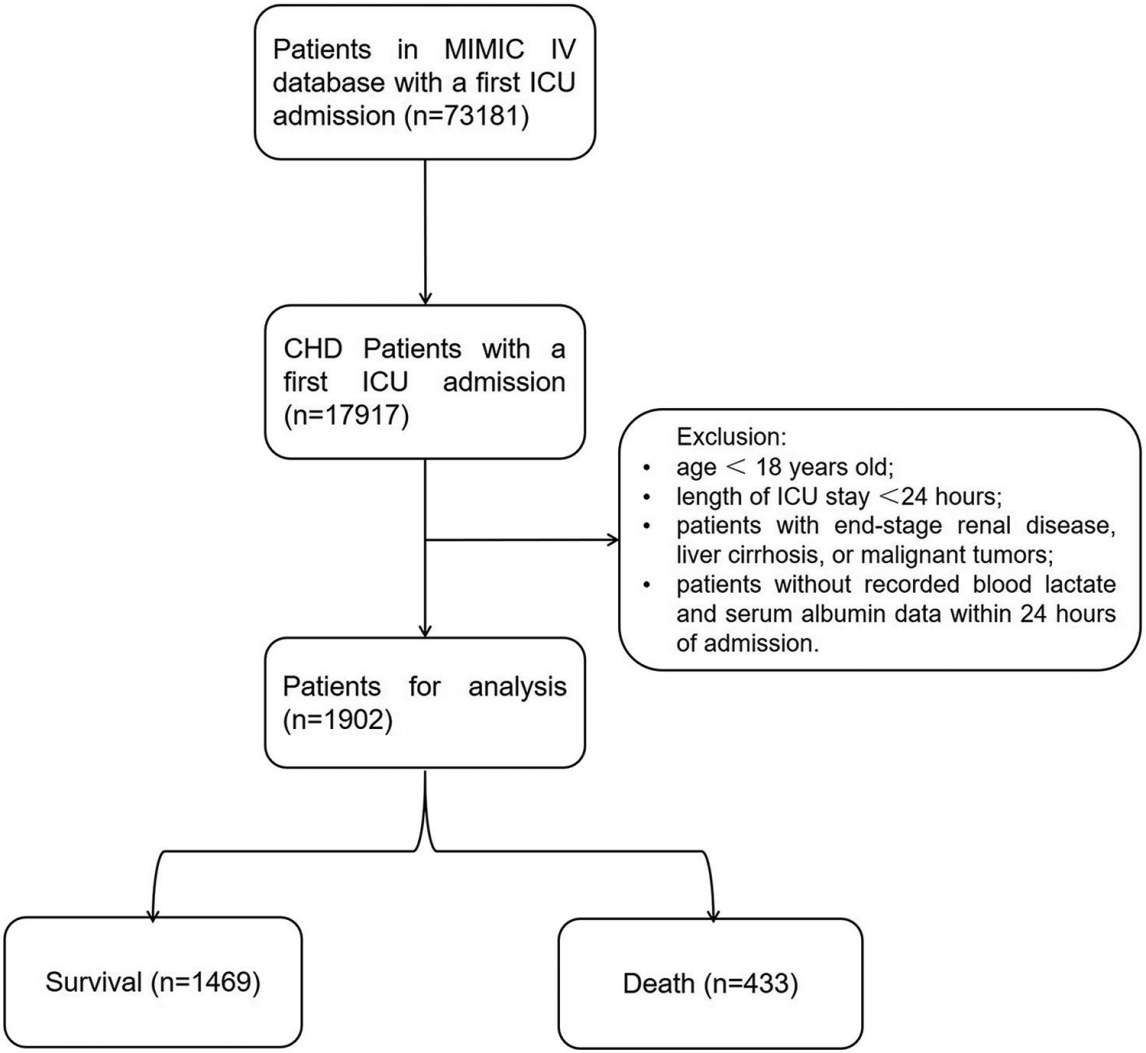
Inclusion and exclusion of patients.

From the MIMIC-IV database (v2.2), our analysis retrospectively included ICU patients diagnosed with CHD (the top 3 ranks) according to the International Classification of Diseases (ICD)-9 codes (410.xx–414.xx) (410.xx: Acute myocardial infarction; 411.xx: Other acute and subacute forms of ischemic heart disease; 412.xx: Old myocardial infarction; 413.xx: Angina pectoris; 414.xx: Other forms of chronic ischemic heart disease) and the ICD-10 codes (I20.x-I22.x, and I25.x) (I20.x: Angina pectoris; I21.x: Acute myocardial infarction; I22.x: Subsequent myocardial infarction; I25.x: Chronic ischemic heart disease). For patients with multiple ICU admissions, only data from the first admission were considered in our analysis. Patients meeting the following criteria will be excluded: (1) patients under 18 years old at the time of initial admission; (2) patients with multiple admissions for CHD, only data from the first admission will be retained; (3) patients with ICU stays of no more than 24 h; (4) patients with end-stage renal disease, liver cirrhosis, or malignant tumors; (5) patients without recorded blood lactate and serum albumin data within 24 h of admission (Figure 1).

### 2.3 Data extraction

We adopted Navicat Premium software (version 16.3.5) and used Structure Query Language (SQL) with PostgreSQL tools (v13.7.1) to extract the raw data of CHD patients within the first day after ICU admission. The data included demographic information (age, gender, race), vital signs (heart rate, SBP, DBP, temperature, respiratory rate), laboratory indicators (PH, PaO2, total CO2, total bilirubin, sodium, potassium, lactate, albumin, PTT, ALT, AST, PT, platelet, RBC, WBC), treatments (CRRT, ventilator), comorbidities (myocardial infarction, paraplegia, diabetes, rheumatic disease, congestive heart failure), and specific scores (SOFA score and GCS score).

For vital signs, laboratory tests, and specific scores that were measured multiple times during the ICU stay, we use the first measurement taken within the first 24 h of ICU admission.

### 2.4 Statistical analysis

Comparison of continuous variables was performed using the *t*-test or Kruskal–Wallis test, with results presented as mean ± standard deviation. Categorical variables were presented as percentages. Kaplan–Meier survival analysis was used to assess the incidence of 28-day mortality events between groups according to different levels of LAR. The hazard ratio (HR) and 95% confidence interval (CI) between LAR and primary endpoints were estimated using Cox proportional hazard models and adjusted for multiple models. Model 1 with no adjustments; Model 2 adjusted for gender, age, and race; and Model 3 adjusted for gender, age, race, pH, PaO2, total CO2, total bilirubin, potassium, PTT, PT, ALT, AST, RBC, WBC, heart rate, SBP, temperature, respiratory rate, SOFA, GCS, Charlson Comorbidity Index, myocardial infarction, paraplegia, rheumatic disease, and CRRT. When LAR was considered as a quartile variable, the lowest quartile was chosen as the reference group. Restricted cubic spline (RCS) Cox regression analysis was further used to determine if there was a potential non-linear relationship between LAR incidence and 28-day mortality in CHD patients, The optimal cut-off value of LAR was determined by the Youden index. Receiver Operating Characteristic (ROC) analysis was used to assess the predictive ability of Lactate, Albumin and LAR for predicting the 28-day mortality. All statistical analyses were performed using R software version 4.3.1. A two-tailed test indicating *P* < 0.05 is considered statistically significant.

## 3 Results

### 3.1 Baseline patient characteristics

A total of 1,902 patients with coronary artery disease meeting the inclusion criteria were included (1,156 females and 746 males), with an average age of 71.62 ± 13.26 years. The results showed statistically significant differences among the quartile groups (Q1–Q4) in age, PH, PaO2, total CO2, SBP, total bilirubin, potassium, lactate, albumin, PPT, PT, ALT, AST, WBC, heart rate, DBP, temperature, respiratory rate, SOFA score, GCS score, charlson comorbidity index, myocardial infarction, diabetes, CRRT, and 28-day mortality rate. Compared with the other three groups, Q4 group exhibited highest 28-day mortality rate, total bilirubin, potassium, lactate, PPT, PT, ALT, AST, WBC, heart rate, and respiratory rate. Q4 group also had higher prevalence of myocardial infarction, diabetes, and increased likelihood of receiving CRRT treatment compared with other three groups. Conversely, PH, PaO2, total CO2, albumin, and SBP levels were lowest in Q4 group. Please see details in Supplementary Table S1.

### 3.2 Association between LAR and 28-day mortality

**Figure 2:**
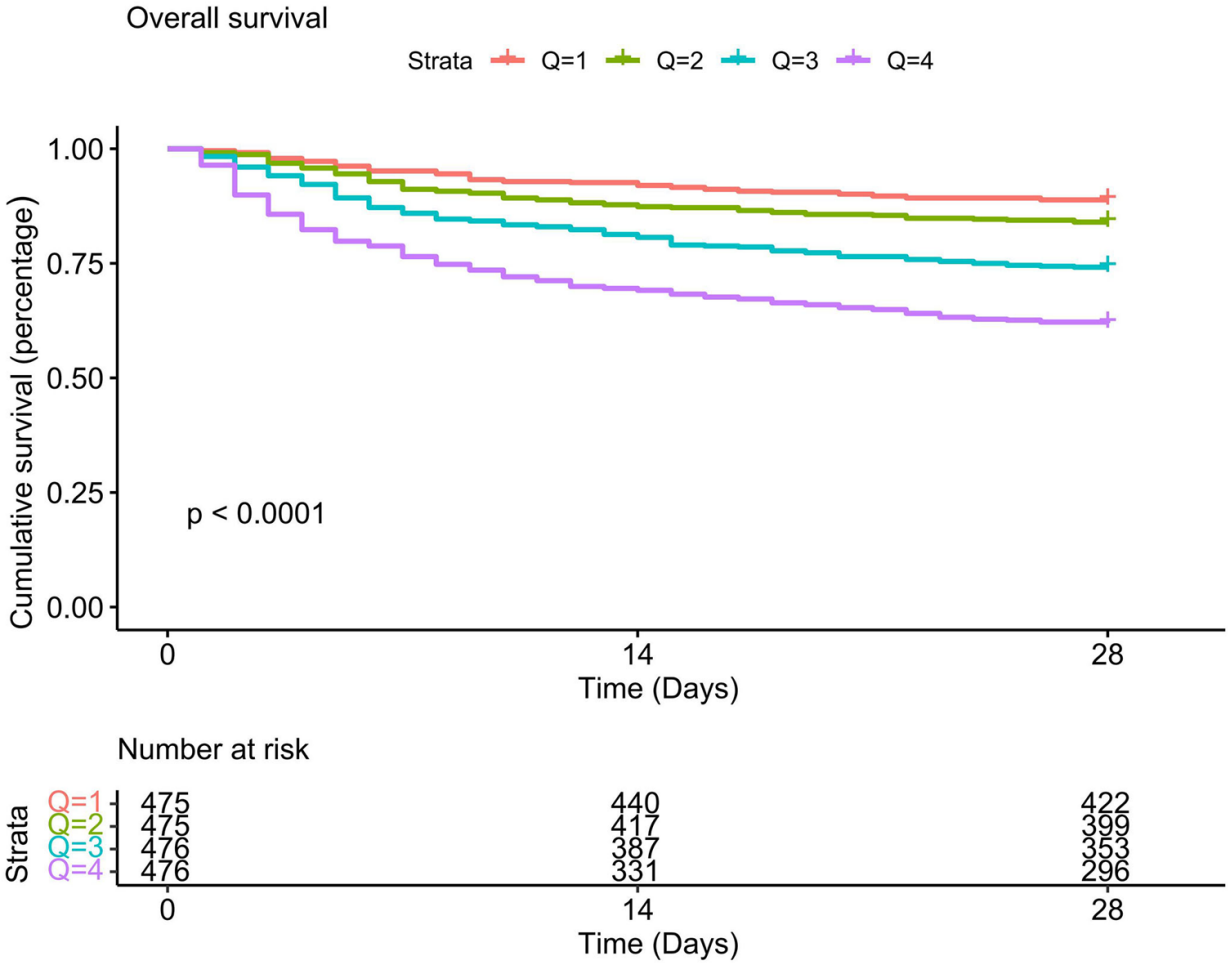
Kaplan-meier survival analysis curve for 28-day mortality.

The Kaplan-Meier survival curve revealed that individuals with high LAR had substantially poor 28-day survival, and the 28-day survival rates of Q1 patients is the highest compared to other quartiles (*P* < 0.01). With the increase of the LAR value, the cumulative 28-day mortality rate of each quartile decreased sequentially (see Figure 2). We further explored the association between the LAR value and 28-day mortality using multivariate Cox proportional hazards regression analyses. To minimize the impact of confounding factors, we constructed three models. In Model1, LAR was positively correlated with 28-day mortality [hazard ratio (HR) = 1.48 (95% CI: 1.39–1.58)]. When LAR was considered as a quartile variable (*P* for trend <0.001), the hazard ratio for 28-day mortality in the highest quartile (Q4) of LAR was 4.14 (95% CI: 3.05–5.63), which was significantly higher than that of Q1 (*P* < 0.001). In Models 2 and 3, we adjusted for different confounding factors (Model II adjusted for race, gender, and age; Model 3 adjusted for gender, age, race, pH, paO2, total CO2, total bilirubin, potassium, partial thromboplastin time, prothrombin time, ALT, AST, WBC, heart rate, respiratory rate, SOFA score, GCS score, Charlson comorbidity index, myocardial infarction, paraplegia, rheumatic disease, and CRRT factors), and this association between the LAR value and 28-day mortality remained the same (see Supplementary Table S2). Besides, results of single factor analysis on the difference analysis of various factors (gender, age, race, pH, paO2, total CO2, total bilirubin, potassium, partial thromboplastin time, prothrombin time, ALT, AST, WBC, heart rate, respiratory rate, SOFA score, GCS score, Charlson comorbidity index, myocardial infarction, paraplegia, rheumatic disease, and CRRT factors) between the death group and the non-death group were shown in Supplementary Table S3.

**Figure 3:**
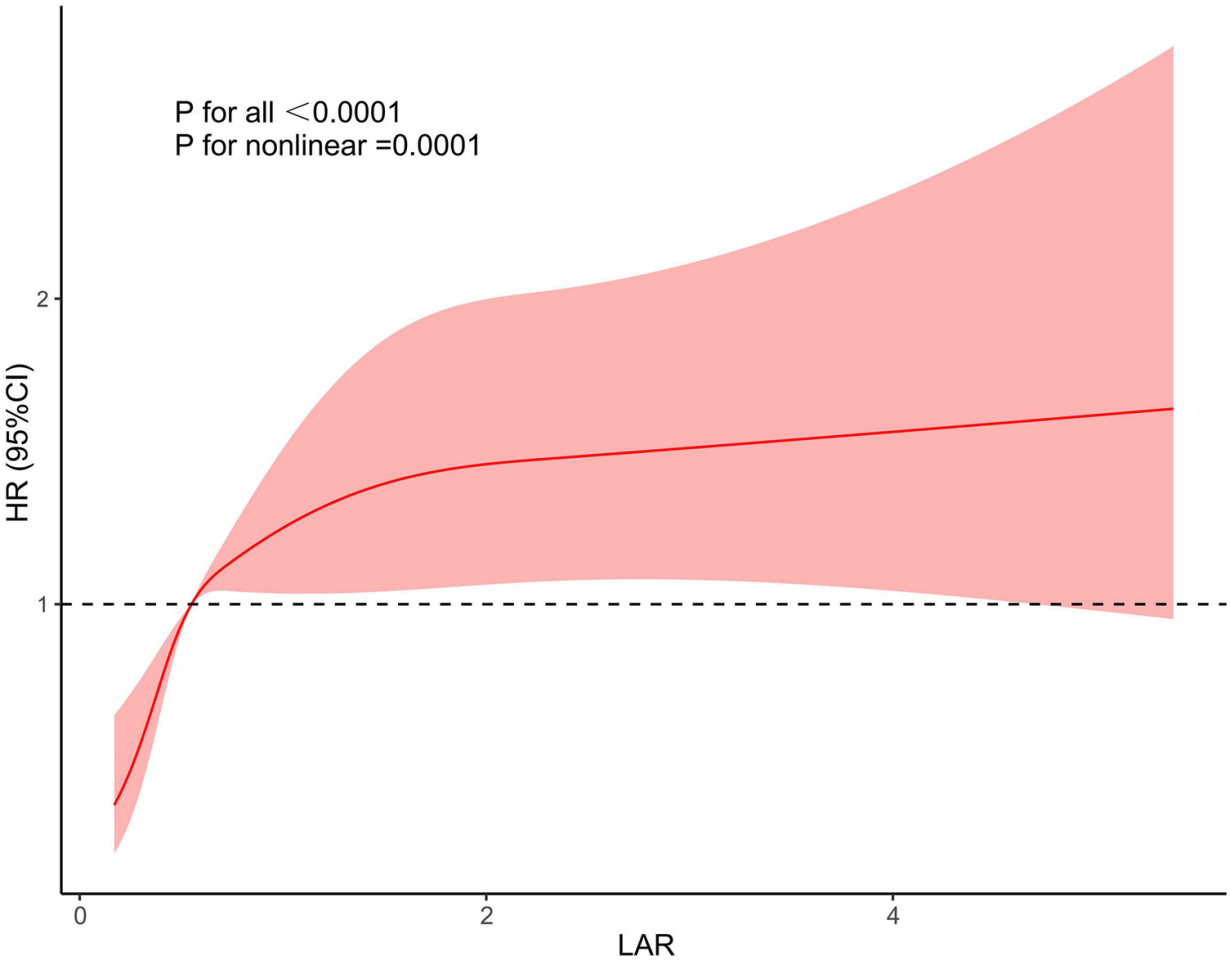
Restricted cubic spline model for association between the LAR and hazard ratio.

We further used restricted cubic splines to further evaluate whether there is a nonlinear relationship between LAR and 28-day mortality in CHD. We found that the relationship between LAR and 28-day mortality in coronary heart disease was approximately L type shaped (Figure 3). After adjusting for confounding factors, when LAR was higher than 0.56, the 28-day mortality in coronary heart disease increased with higher LAR value.

### 3.3 ROC curve analysis and prediction of mortality

**Figure 4:**
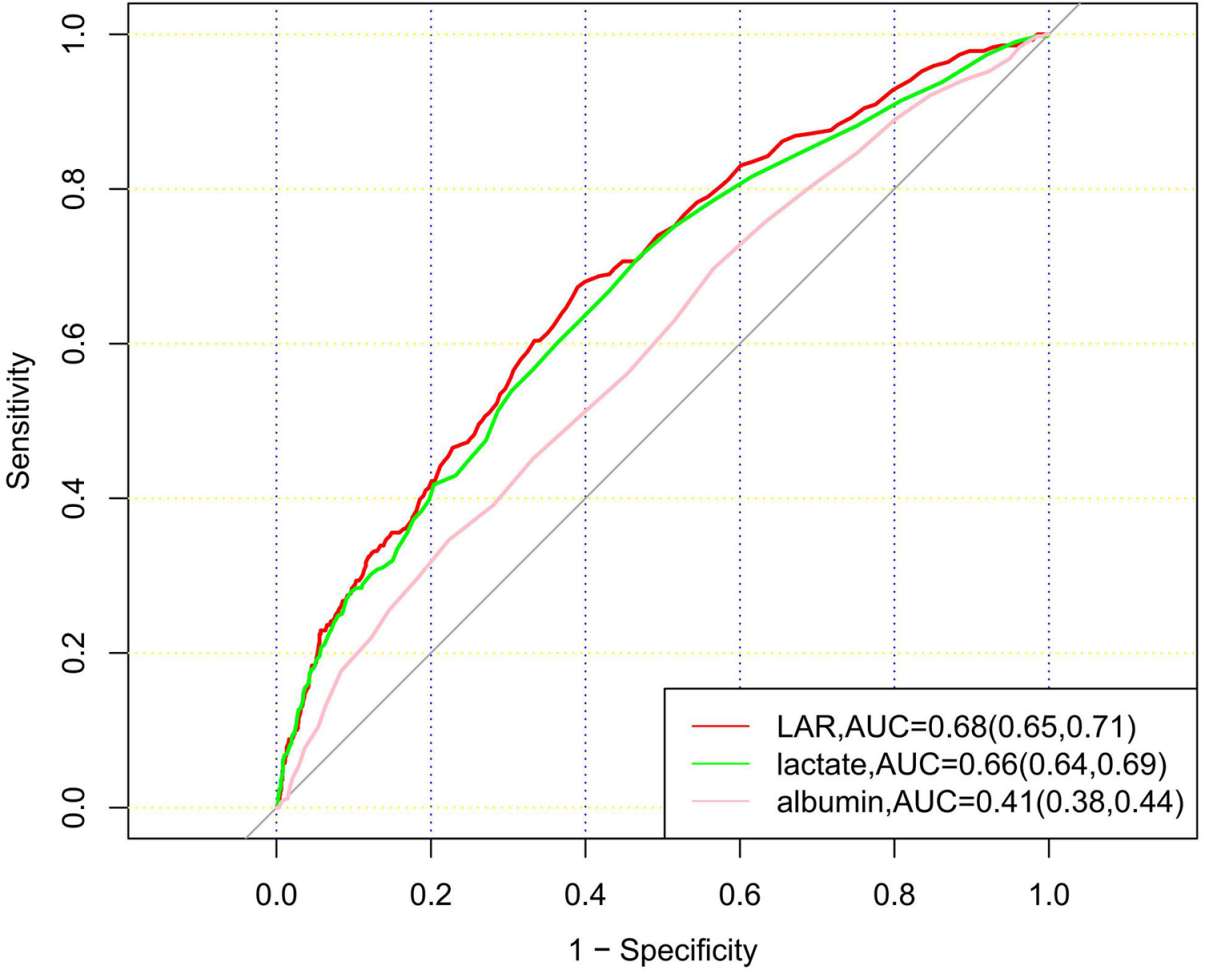
ROC curves for 3 indicators, LAR, lactate and albumin, to predict mortality.

ROC curves for LAR, albumin, and lactate were plotted to predict the 28-day mortality of patients with CHD (Figure 4). The results showed that the area under the curve (AUC) of LAR was 0.68 (95% CI: 0.65–0.71), which was superior to that of lactate 0.66 (95% CI: 0.64–0.69) and albumin 0.41 (95% CI: 0.38–0.44). The results showed that LAR was more effective in predicting 28-day mortality of CHD patients than albumin or lactate alone.

### 3.4 Subgroup analysis

Subgroup analysis was used to assess the association between LAR and 28-day mortality of CHD patients (Table 1). When stratified for age, gender, race, paraplegia, myocardial infarct, rheumatic disease, CRRT. The results showed no significant interaction between LAR and most subgroups (*P* for interaction > 0.05). However, an interaction was observed between race and CRRT subgroups (*P* for interaction < 0.05).

**Table 1:** Subgroup analysis.

Variables	HR (95% CI)	*P* for interaction
Age		0.415
≥65	1.14 (1.01, 1.28)	
<65	1.21 (0.95, 1.54)	
Gender		0.346
Female	1.05 (0.91, 1.20)	
Male	1.41 (1.17, 1.70)	
Race		0.004
White	1.30 (1.12, 1.50)	
Other	1.08 (0.92, 1.28)	
Paralegia		0.25
Yes	1.14 (1.03, 1.27)	
No	6.84 (1.32, 35.46)	
Myocardial infarct		0.721
Yes	1.11 (0.90, 1.37)	
No	1.17 (1.04, 1.33)	
Rheumatic disease		0.07
Yes	2.16 (0.61, 7.67)	
No	1.14 (1.03, 1.27)	
CRRT		0.003
Yes	1.22 (0.95, 1.58)	
No	1.21 (1.06, 1.37)	

## 4 Discussion

Our study used the publicly accessible MIMIC-IV database (v2.2) to assess the prognostic values of LAR level in patients with CHD. This retrospective analysis demonstrated a measurable association between elevated LAR levels and increased 28-day mortality in CHD patients. KM survival analysis indicated that patients with higher LAR levels had increased risk of death within 28 days of admission compared to those with lower LAR levels. The RCS curve revealed that when the LAR value exceeded 0.56, the mortality rate increased with rising LAR values. Subgroup analysis confirmed that the LAR level could serve as an independent predictor of 28-day mortality. Moreover, ROC curve analysis demonstrated that LAR had superior predictive performance for 28-day mortality compared to lactate or albumin alone.

### 4.1 The relationship between serum lactate, albumin, LAR and cardiovascular diseases

Lactate is a product of anaerobic metabolism, and it occurs when the body is under anoxic condition, leading to an increase in glycolysis. Elevated lactate levels reflect tissue hypoxia, metabolic disorders, and systemic inflammation, all of which are closely related to cardiovascular diseases (11, 12). Albumin is a protein synthesized by the liver, which plays a crucial role in maintaining blood osmotic pressure and transporting various substances such as fatty acids, hormones, and drugs. Albumin levels reflect nutritional status and liver function, and are also associated with inflammatory responses. Multiple studies have revealed that lactate and albumin can serve as prognostic and mortality predictors for cardiovascular diseases. Studies have found that elevated serum lactate levels and low albumin levels in patients with acute coronary syndrome (ACS) are associated with poorer outcomes, increased in-hospital and long-term mortality rates (8, 13, 14), indicating that lactate and albumin levels can serve as an effective predictor of prognosis in ACS patients. The serum lactate levels are also important prognostic indicators in heart failure patients. Heart failure patients with elevated lactate levels have a significantly increased risk of death both during hospitalization and after discharge (7, 15, 16). Besides, patients with low albumin levels have higher mortality rates during hospitalization and long-term follow-up (6, 17). In cardiac surgery, such as coronary artery bypass grafting or valve replacement, monitoring serum lactate levels help predict postoperative complications and mortality risk (18, 19). Persistent elevation of lactate levels and low albumin levels after surgery usually indicates a higher incidence of complications and poorer survival rates (19–22). The serum lactate and albumin levels in patients with cardiogenic shock are closely related to the severity of their condition and prognosis. Patients with high lactate levels generally have poorer prognosis and higher mortality rates (23, 24).

LAR reflect the metabolic load and nutritional status of patients, thereby providing a more comprehensive prognostic assessment. Numerous studies have demonstrated that LAR is more advantageous in predicting prognostic indicators such as mortality and complication rates in critical patients compared to relying solely lactate or albumin levels. High LAR values are often associated with poorer prognosis, making it a more sensitive and specific prognostic indicator (14, 25, 26). Additionally, LAR helps in better risk stratification, aiding clinicians in identifying high-risk patients for timely and targeted interventions. For patients with high LAR values, enhanced monitoring and more aggressive treatment strategies may be warranted.

LAR is an important indicator for predicting mortality in critical patients (27). It has shown good effectiveness in predicting prognosis and mortality in patients with cardiovascular diseases such as heart failure, myocardial infarction, and cardiac arrest. Studies have indicated that high LAR levels are independent risk factors for high mortality rates in patients with severe heart failure (28, 29). LAR can also predict the risk of readmission within three months for heart failure patients (30). Yang et al. (31) analyzed 2,816 hospitalized patients diagnosed with myocardial infarction and found that the LAR value was significantly higher in the death group compared to the survival group, demonstrating a significant independent predictive ability for increased all-cause mortality during hospitalization. Another study showed that the LAR ratio is significantly associated with 14-day, 28-day, and 90-day all-cause mortality in critical AMI patients, with higher LAR being considered an independent risk factor for higher mortality in AMI patients (31). Haschemi et al. (32) demonstrated that LAR could predict 30-day survival after in-hospital cardiac arrest, with better prognostic performance for predicting 30-day survival compared to lactate or albumin alone. Additionally, LAR is significantly associated with poor prognosis in HF and CKD patients, and it may help identify HF and CKD patients with a high risk of all-cause mortality (33). Our study systematically revealed that the higher the LAR value, the higher the 28-day mortality rate with CHD patients, which will provide guidance for clinical treatment.

### 4.2 Mechanisms of increased LAR levels in CHD patients

CHD is often accompanied by coronary artery stenosis or obstruction, leading to decreased oxygen supply to myocardial cells. In hypoxic conditions, myocardial cells cannot produce enough energy through aerobic metabolism (oxidative phosphorylation) and instead rely on anaerobic metabolism (glycolysis) to generate energy. The end product of glycolysis is lactate, lactate accumulates and is released into the blood under ischemic conditions, leading to elevated blood lactate levels. Additionally, the ability of myocardial cells to clear lactate may also decrease, further contributing to the accumulation of lactate in the blood. Coronary artery disease patients often have endothelial dysfunction, which can reduce coronary blood flow and further decrease myocardial oxygen supply. Endothelial dysfunction may also affect lactate clearance and metabolism, which leads to increased blood lactate levels. Moreover, coronary artery disease patients often accompany with activation of the sympathetic nervous system, which increases the metabolic demand of the myocardium. When oxygen supply is insufficient, myocardial cells rely more on anaerobic metabolism, thus producing more lactate.

Liver is the main organ for synthesizing albumin. Patients with coronary artery disease may have mild to moderate liver function abnormalities, especially those with other chronic diseases (such as liver cirrhosis, hepatitis) or those using certain medications (such as statins). These factors can affect the liver's protein synthesis function, leading to reduced albumin synthesis. Furthermore, patients with coronary artery disease may have insufficient nutritional intake due to decreased appetite and gastrointestinal dysfunction, which can reduce the synthesis of plasma proteins (including albumin), leading to lower albumin levels. Coronary artery disease can also cause heart failure, which lead to hepatic congestion and renal insufficiency, thereby impacting albumin metabolism and contributing to decreased albumin levels.

Among our 1,902 CHD patients, primarily including those with myocardial infarction, ischemic heart disease, and angina, the possible mechanisms contributing to a higher LAR (lactate/albumin ratio) are as follows: In myocardial infarction and patients, insufficient blood supply to the heart prompts myocardial cells to undergo anaerobic metabolism, thus increasing lactate production. Besides, heart disease patients often accompany with renal dysfunction, which affects albumin metabolism and leads to decreased levels. During angina episodes, the temporary insufficiency of blood supply to the myocardium leads to an increase in anaerobic metabolism and a rise in lactate production. Prolonged ischemic conditions can impair the metabolic function of myocardial cells, resulting in lactate accumulation.

### 4.3 Study limitations

Despite significant progress in research on lactate as a prognostic and mortality predictor for cardiovascular diseases, there are still some limitations. Firstly, since the patients in our study were all admitted to the ICU, there were no stable CHD patients, which may result in our conclusions not being applicable to CHD patients in the general ward. Secondly, differences in lactate measurement methods and timing across studies may limit the comparability of results. What is more, more large-scale, multicenter prospective studies are needed to validate the predictive value of the LAR in coronary heart disease populations.

## 5 Conclusions

Our study demonstrated that the LAR value can be an important risk predictor of 28-day mortality in patients with CHD, and a higher LAR associate with increased mortality rate.

## Data Availability

The datasets presented in this study can be found in online repositories. The names of the repository/repositories and accession number(s) can be found below: https://physionet.org/content/mimiciv/2.2/.
