# Supplementary material for: Association between lactate/albumin ratio and 28-day mortality in ICU critical patients with coronary heart disease: a retrospective analysis of the MIMIC-IV database
Source: Front Cardiovasc Med. 2024 Nov 18;11:1486697. doi: 10.3389/fcvm.2024.1486697 (PMC11609210; doi:10.3389/fcvm.2024.1486697)
Supplement: Supplementary file 1 [file Table1.pdf]

Supplementary Table 1. Baseline patient characteristics according to the lactate/albumin ratio

Variables	Total n = 1902	Lactate/albumin ratio				P value
		Q1 (n=475)	Q2 (n=475)	Q3 (n=476)	Q4 (n=476)	
LAR	0.83±0.84	0.29 ±0.06	0.46 ±0.05	0.71 ±0.10	1.86±1.13	<0.001
Age, years	71.62±13.26	70.82 ±12.86	71.74±12.08	71.28 ±14.12	72.65±13.85	0.177
Female (%)	1156 (60.8)	306 (64.4)	298 (62.7)	280 (58.8)	272 (57.1)	0.078
Race, white (%)	664 (34.9)	150 (31.6)	170 (35.8	161 (33.8)	183 (38.4)	0.148
**Laboratory Information**						
PH	7.34 ±0.11	7.37 ±0.08	7.37±0.09	7.35±0.10	7.28 ±0.13	<0.001
PaO2	141.95±123.87	176.37 ±138.57	145.43±122.78	128.94±118.21	117.14 ±106.06	<0.001
Total CO2	24.05 ±6.00	25.90 ±5.59	25.62±5.75	24.07±5.76	20.63±5.41	<0.001
Total bilirubin	1.00 ±1.55	0.75 ±0.88	0.89±1.41	1.05±1.36	1.29 ±2.19	<0.001
Sodium	138.45 ±5.62	138.38 ±4.26	138.86±5.11	138.14±6.54	138.41 ±6.26	0.256
Potassium	4.37±0.84	4.30 ±0.81	4.28±0.78	4.40±0.84	4.50 ±0.91	<0.001
Lactate	2.42±2.11	1.00 ±0.22	1.47±0.29	2.16±0.53	5.05 ±2.72	<0.001
Albumin	3.15±0.63	3.49 ±0.52	3.22±0.55	3.07±0.62	2.83 ±0.64	<0.001
PTT	46.56±32.94	46.82 ±33.32	43.71±29.63	46.91±33.93	48.76 ±34.51	0.135
PT	17.82±12.12	15.76 ±10.19	16.58±9.34	17.86±11.63	21.02 ±15.62	<0.001
ALT	174.75 ±595.74	87.14 ±420.39	80.56±244.88	181.34±652.16	345.75 ±839.48	<0.001
AST	316.17±1375.48	135.56 ±633.13	117.00 ±400.99	330.42±1510.36	670.98 ±2110.35	<0.001
Platelet	216.66 ±106.73	216.38 ±94.94	219.95 ±102.98	212.21±104.06	218.13 ±123.10	0.712
RBC	3.72 ±0.79	3.69 ±0.77	3.67±0.74	3.79±0.82	3.72 ±0.84	0.077
WBC	13.95 ±7.50	11.06 ±5.51	13.32 ±6.70	15.43 ±7.52	15.99 ±8.84	<0.001
**Vital signs**						
Heart rate	88.76 ±21.06	81.69±18.36	86.58 ±21.04	90.96±20.33	95.78 ±21.78	<0.001
SBP	121.73 ±24.56	125.63±24.44	123.65±24.33	120.47±24.61	117.17 ±24.10	<0.001
DBP	67.53±19.26	67.31±19.39	67.28±18.15	69.18 ±20.65	66.38 ±18.73	0.15
Body temperature	36.66±0.92	36.67±0.84	36.74±0.82	36.70 ±0.93	36.52 ±1.07	0.002
Resp rate	20.20±6.17	18.50±5.48	19.97±5.99	20.45 ±6.24	21.90 ±6.45	<0.001
**Scoring systems**						
SOFA score	2.43±2.48	1.73±2.01	2.20 ±2.30	2.34±2.25	3.43±2.95	<0.001
GCS score	14.33±2.14	14.52±1.79	14.28 ±2.24	14.39±1.95	14.14±2.50	0.046
**Comorbidities**						
Charlson comorbidity index	6.06±2.49	5.71 ±2.48	5.94 ±2.36	6.17±2.53	6.42 ±2.55	<0.001
Myocardial infarct (%)	1105 (58.1)	248 (52.2)	276 (58.1)	280 (58.8)	301 (63.2)	0.007
Paraplegia (%)	72 (3.8)	23 (4.8)	19 (4.0)	20 (4.2)	10 (2.1)	0.142
Diabetes (%)	795 (41.8)	171 (36.0)	203 (42.7)	209 (43.9)	212 (44.5)	0.028
Rheumatic disease (%)	98 (5.2)	30 (6.3)	19 (4.0)	25 (5.3)	24 (5.0)	0.453
Congestive heart failure (%)	1050 (55.2)	242 (50.9)	268 (56.4)	272 (57.1)	268 (56.3)	0.193
**Treatment**						
CRRT (%)	136 (7.2)	13 ((2.7)	19 (4.0)	30 (6.3)	74 (15.5)	<0.001
Ventilator (%)	1719 (90.4)	439 (92.4)	432 (90.9)	424 (89.1)	424 (89.1)	0.229
28-day outcome (%)	433 (22.8)	53(11.2)	76 (16.0)	123 (25.8)	181 (38.0)	<0.001

PaO2, Partial oxygen pressure; Total CO2, Total carbon dioxide; PTT, Partial thromboplastin time; PT, Prothrombin time; ALT, Alanine aminotransferase; AST, Aspartate aminotransferase; RBC, Red Blood Cell Count; WBC, White Blood Cell; SBP, Systolic blood pressure; DBP, Diastolic blood pressure; Resp rate, respiratory rate; SOFA, Sequential Organ Failure Assessment; GCS, Glasgow Coma Scale; CRRT, Continuous renal replacement therapy.
