# Supplementary material for: Association between lactate/albumin ratio and 28-day mortality in ICU critical patients with coronary heart disease: a retrospective analysis of the MIMIC-IV database
Source: Front Cardiovasc Med. 2024 Nov 18;11:1486697. doi: 10.3389/fcvm.2024.1486697 (PMC11609210; doi:10.3389/fcvm.2024.1486697)
Supplement: Supplementary file 2 [file Table2.pdf]

Supplementary Table 2. Hazard ratios and 95% confidence intervals for 28-day mortality based on lactate/albumin ratio quartiles

	**Model1**		**Model2**		**Model3**	
	**HR (95%CI)**	**P**	**HR (95%CI)**	**P**	**HR (95%CI)**	**P**
LAR	1.48 [1.39, 1.58]	<0.001	1.46 [1.37, 1.56]	<0.001	1.16 [1.04, 1.28]	0.006
Q1	Reference		Reference		Reference	
Q2	1.47 [1.04, 2.09]	0.030	1.41 [1.00, 2.01]	0.03	1.38 [0.94, 2.02])	0.100
Q3	2.52 [1.83, 3.48]	<0.001	2.46 [1.78, 3.39]	<0.001	2.04 [1.42, 2.92]	<0.001
Q4	4.14 [3.05, 5.63]	<0.001	3.86 [2.84, 5.25]	<0.001	2.18 [1.50, 3.18]	<0.001
**P for trend**	<0.001		<0.001		<0.001	

Model1: Unadjusted model

Model2: Adjusted for age and race

Model3: Adjusted for gender, age, race, pH, paO2, total CO2, total bilirubin, potassium, PTT, PT, ALT, AST, WBC, heart rate, resp rate, SOFA score, GCS score, charlson comorbidity index, myocardial infarct, paraplegia, rheumatic disease, CRRT
