# Supplementary material for: Association between lactate/albumin ratio and 28-day mortality in ICU critical patients with coronary heart disease: a retrospective analysis of the MIMIC-IV database
Source: Front Cardiovasc Med. 2024 Nov 18;11:1486697. doi: 10.3389/fcvm.2024.1486697 (PMC11609210; doi:10.3389/fcvm.2024.1486697)
Supplement: Supplementary file 3 [file Table3.pdf]

Supplementary Table 3. Results of single factor analysis

	Survival（n=1469）	Survival（n=433）	**P**
LAR	0.72 ±0.70	1.21 ±1.12	<0.001
**Demographic data**			
Age, years	70.44 ±13.34	75.62 ±12.17	<0.001
Female (%)	549 (37.4)	197 (45.5)	0.003
Race, white (%)	984 (67.0)	254 (58.7)	0.002
**Laboratory Information**			
PH	7.35 ±0.10	7.31 ±0.12	<0.001
PaO2	150.03 ±128.40	114.53 ±102.56	<0.001
Total CO2	24.41 ±5.62	22.82 ±7.02	<0.001
Total bilirubin	0.95 ±1.52	1.16 ±1.65	0.017
Sodium	138.34 ±5.41	138.80 ±6.29	0.136
Potassium	4.34 ±0.83	4.47 ±0.87	0.005
Lactate	2.14 ±1.76	3.39 ±2.78	<0.001
Albumin	3.20 ±0.63	2.99 ±0.63	<0.001
PTT	45.57 ±31.88	49.88 ±36.09	0.018
PT	16.99 ±11.35	20.58 ±14.07	<0.001
ALT	153.54 ±550.60	246.40 ±724.00	0.005
AST	263.73 ±1212.01	492.55 ±1811.33	0.003
Platelet Count	214.97 ±102.54	222.42 ±119.83	0.202
RBC	3.74 ±0.79	3.64 ±0.79	0.029
WBC	13.31 ±7.07	16.14 ±8.43	<0.001
**Vital signs**			
Heart rate	87.81 ±20.86	91.94 ±21.43	<0.001
SBP	122.49 ±24.65	119.14 ±24.11	0.013
DBP	67.84 ±19.49	66.49 ±18.47	0.202
Body temperature	36.72 ±0.83	36.44 ±1.15	<0.001
Resp rate	19.75 ±5.97	21.73 ±6.59	<0.001
**Scoring systems**			
SOFA score	2.25 ±2.36	3.03 ±2.78	<0.001
GCS score	14.40 ±1.98	14.10 ±2.60	0.01
**Comorbidities**			
Charlson comorbidity index	5.79 ±2.41	6.98 ±2.55	<0.001
Myocardial infarct (%)	830 (56.5)	275(63.5)	0.011
Paraplegia (%)	46 (3.1)	26 (6.0)	0.009
Diabetes (%)	609 (41.5)	186 (43.0	0.617
rheumatic_disease (%)	66 (4.5)	32 (7.4)	0.023
congestive_heart_failure (%)	797 (54.3)	253 (58.4)	0.139
**Treatment**			
CRRT (%)	63 (4.3)	73 (16.9)	<0.001
ventilator (%)	1338 (91.1)	381 (88.0)	0.068
